# First mussel settlement observed in Antarctica reveals the potential for future invasions

**DOI:** 10.1038/s41598-020-62340-0

**Published:** 2020-03-26

**Authors:** Leyla Cárdenas, Jean-Charles Leclerc, Paulina Bruning, Ignacio Garrido, Camille Détrée, Alvaro Figueroa, Marcela Astorga, Jorge M. Navarro, Ladd E. Johnson, James T. Carlton, Luis Pardo

**Affiliations:** 1 Centro FONDAP de Investigación en Dinámica de Ecosistemas Marinos de Altas Latitudes (IDEAL), Valdivia, Chile; 2 Instituto de Ciencias Ambientales y Evolutivas, Facultad de Ciencias, Universidad Austral de Chile, Valdivia, Chile; 3 Universidad Católica de la Santísima Concepción, Centro de Investigación en Biodiversidad y Ambientes Sustentables (CIBAS), Concepción, Chile; 4 Instituto de Acuicultura, Universidad Austral de Chile, Puerto Montt, Chile; 5 Instituto Ciencias Marinas y Limnológicas, Facultad de Ciencias, Universidad Austral de Chile, Valdivia, Chile; 6 Department of Biology and Quebec-Ocean Institute, Laval University, Québec, QC Canada; 7 Maritime Studies Program, Williams College, Mystic Seaport, Mystic, CT USA

**Keywords:** Ecology, Climate sciences, Ecology, Environmental sciences, Natural hazards, Ocean sciences

## Abstract

Global biodiversity is both declining and being redistributed in response to multiple drivers characterizing the Anthropocene, including synergies between biological invasions and climate change. The Antarctic marine benthos may constitute the last biogeographic realm where barriers (oceanographic currents, climatic gradients) have not yet been broken. Here we report the successful settlement of a cohort of *Mytilus* cf. *platensis* in a shallow subtidal habitat of the South Shetland Islands in 2019, which demonstrates the ability of this species to complete its early life stages in this extreme environment. Genetic analyses and shipping records show that this observation is consistent with the dominant vectors and pathways linking southern Patagonia with the Antarctic Peninsula and demonstrates the potential for impending invasions of Antarctic ecosystems.

## Introduction

Planetary changes during the Anthropocene have led to an unprecedented rate of biodiversity declines and redistribution, in response to the interplay of drivers such as climate change and species invasions^1–3^. As the list of endangered and extinct species increases, so does the list of emerging invaders in response to expanding and intensifying pathways from new donor and source regions^4^. These planetary changes redefine biogeographic boundaries and strengthen a longstanding pattern of increasing biotic homogenization^5,6^.

Despite the ongoing homogenization of the planet’s flora and fauna^4^, some biogeographic regions have so far been relatively free from recent invasions. The most obvious is Antarctica where a large fraction of the indigenous biota has diverged from other realms due to poleward tectonic drift, exacerbated by climate and altered oceanographic boundaries^7–9^. In contrast to a depauperate terrestrial realm, Antarctic marine waters are inhabited by a rich and mostly endemic biota (>50%), which has experienced a massive diversification over the last 50 million years^8,9^ with increasing isolation until *circa* 5 million years ago due to the formation of the Antarctic Polar Front (APF), a major marine biogeographic boundary^10,11^. This isolation is notably supported by the fact that nearshore and continental shelf benthic assemblages of the Southern Ocean show clear genetic singularities with their northern counterparts^11^.

The shallow Antarctic benthos has remained uninvaded thus far^12^ due to the combination of geographical distance (>1000 km), oceanographic circulation (e.g., APF) and environmental conditions (e.g., consistently near-freezing temperatures). Both dispersal and physiological barriers to the establishment of non-indigenous species (NIS) may thus explain why, to date, no known NIS have become established there^12^. Whereas a few non-indigenous invertebrates have been occasionally found in the field, these records have been limited to observations of one or two adult individuals, colonies, or several larval stages, none of which have been known to successfully settle and establish, a necessary step in the invasion process^12,13^. However, as human activity is increasing in Antarctica, human-mediated transport of propagules is occurring^14,15^ and shipping is undoubtedly the primary vector by which this dispersal is occurring^16^. For benthic organisms, this involves either the transport of pelagic larval stages in ballast water or adults fouling hulls or other outer surfaces. Indeed, the known NIS observed in Antarctica have been limited to organisms with traits conducive to these mechanisms of transport: decapod larvae in ballast water and fouling species (e.g., bryozoans, hydroids and tunicates) on ship hulls or kelp rafts^12,13^. Transport alone is not, however, sufficient for the establishment of a NIS, which minimally requires further development in the case of larval stages, settlement and subsequent reproduction. Physical conditions, especially low temperatures, will present a significant challenge to any NIS arriving to Antarctica, but in the context of global climate change, these environmental filters^17^ are likely to become less of a barrier to Antarctic invasibility now and in the future. Such changes are particularly severe in the Antarctic peninsula and surrounding archipels^18^, where human activities (and associated propagule pressures) are concentrated and increasing^12^. In this particular region notably, there is a mounting, critical and robust interest in the discovery of any evidence that the invasion process in the Antarctic has begun^19^.

Here we document the occurrence of juvenile mussels, *Mytilus,* that successfully settled on biogenic substrata in 2019 at subtidal sites within Fildes Bay (also known as Maxwell Bay) in the South Shetland Islands, Antarctica, where there are no native congeneric mussels. Due to their small size, identification required molecular barcoding, which showed that these mussels belong to a clade endemic to southern Patagonia (*Mytilus* cf. *platensis*), the region from which most ship traffic originates.

## Results

### Field sampling

**Figure 1:**
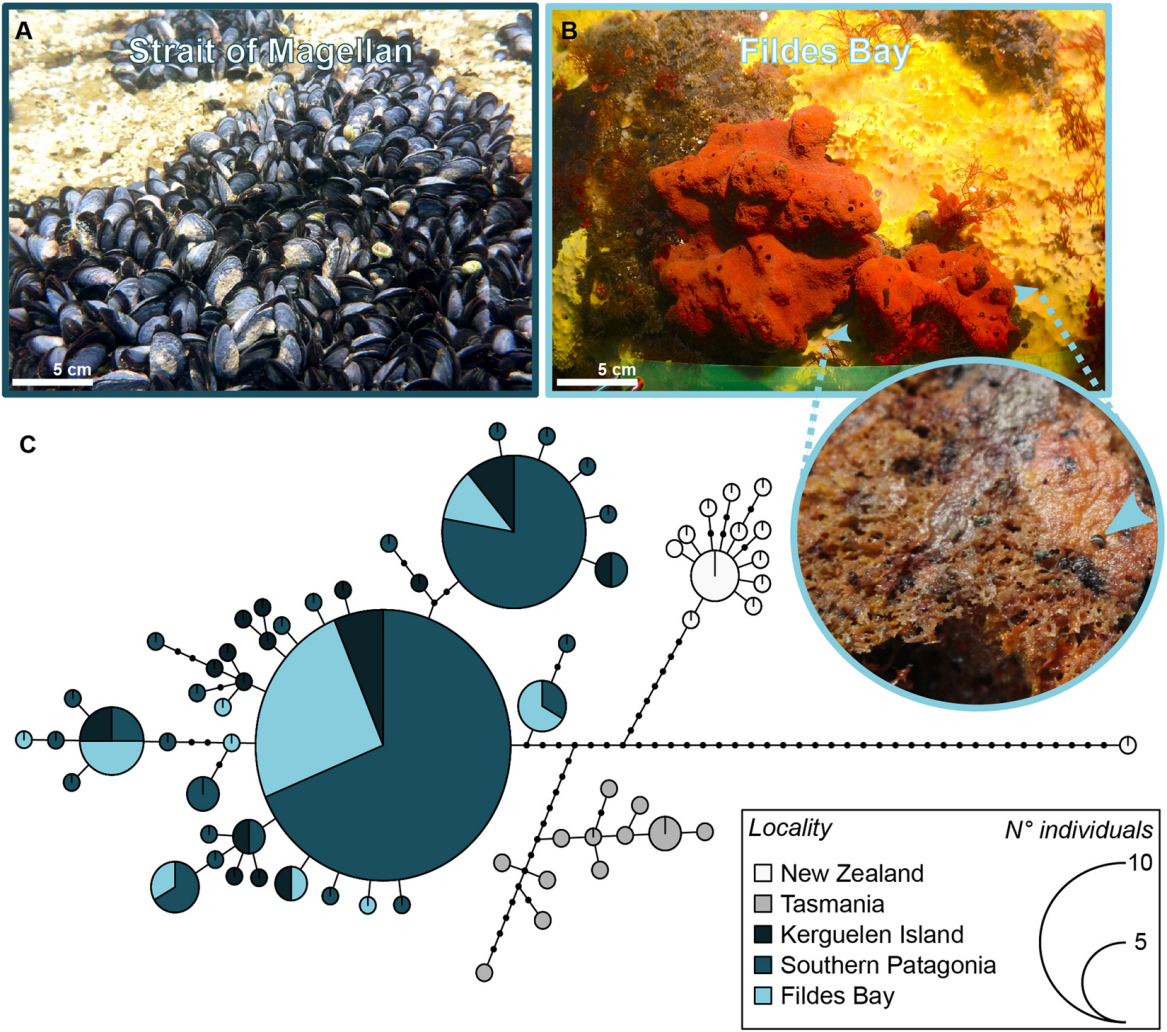
*Mytilus* spp. in the Southern Hemisphere. Mussels may form dense aggregations in the intertidal zone of their putative native range (**A**), such as the Strait of Magellan (Southern Patagonia). Recently detected in Antarctica (Fildes Bay, Southern Shetland Islands), small recruits (highlighted by the blue arrow) were found in biogenic subtidal habitats, such as the endemic sponge *Kirkpatrickia variolosa* (**B**). Within the Southern Hemisphere mitochondrial lineage of *Mytilus spp*. (previously discriminated, Fig. S1), the haplotype network (**C**) based on cytochrome oxidase I gene assigns the specimens collected in Fildes bay to the Southern Patagonian clade of *Mytilus* spp. (i.e. *Mytilus* cf. *platensis*), which had successfully invaded Kerguelen in the late Tertiary era^20^. Each haplotype is represented by a circle with its size proportional to the number of individuals bearing the haplotype over the whole data set. Colours indicate the location of the haplotype and dots correspond to mutational steps among haplotypes. Sequences used for this analysis can be found in the Table S2.

A total of 47 juvenile mussels were found in four of the 75 samples collected from the two study sites in Fildes Bay. Whereas mussels were observed at both sites, virtually all individuals (44) were collected in a single core dominated by the sponge *Kirkpatrickia variolosa* (Kirkpatrick, 1907), and more specifically within its interior cavities (Fig. 1, the sponge identification was done using barcoding). All mussels were small and uniform in size (2.0 mm ± 0.1 mm [mean ± SD]).

### Mussel identification

The phylogenetic analyses were consistent for both COI and 16S (Fig. 1). The topology of the maximum-likelihood (ML) tree was identical to that of the neighbor-joining  distance (NJ) tree as far as nodes supported by bootstrap scores over 50% are concerned (Fig. S1). In general, three clades show robust bootstrap support in both tree reconstructions. Samples mainly from the Southern Hemisphere are in one well-supported clade, which matches previous findings^20^. This clade has higher bootstrap values in the COI tree than in the 16S tree and contained samples of *Mytilus* sp. from South America, Australia, New Zealand, Tasmania and the Kerguelen Islands as well as those from Antarctica (Fig. S1). The haplotype network (Fig. 1) reveals that Antarctic samples and those from South America and the Kerguelen Islands were closely related with a network composed by two common and shared haplotypes separated by few mutational steps. Clade S1, following Gérard *et al*. (2008), did not share haplotypes with New Zealand or Tasmanian samples and was separated from them by 14 and 9 mutational steps, respectively.

### Vector activity and pathways

**Figure 2:**
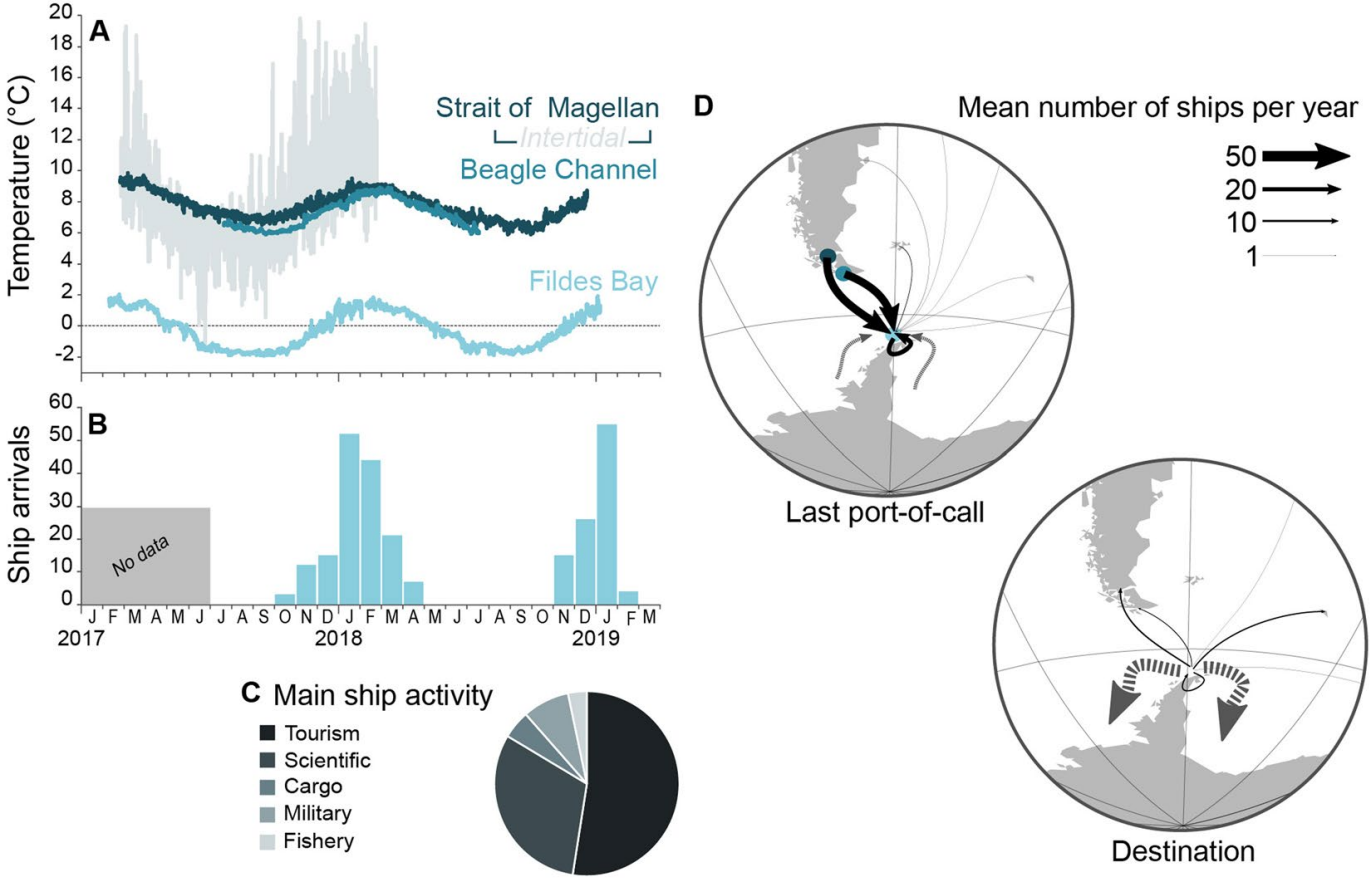
Barriers and pathways to species introduction in Antarctica. Subtidal temperatures recorded over 2017–2019 display a continuous difference of ca. 7–8 °C between Southern Patagonia (Strait of Magellan and Beagle Channel) and Southern Shetlands (Fildes Bay), but intertidal assemblages from southern Patagonia occasionally experience temperature in the range of that of the South Shetland Islands (**A**). Shipping traffic (expressed as the number of arrivals in Fildes Bay per month) peaks in summer (December-February) (**B**). Their main activity is indicated (**C**), along with their last port-of-call and next destination (**D**). Dotted lines indicate undefined origin or destination in Antarctica, including the Antarctic peninsula. Ship activity data courtesy of the Chilean navy.

Over two years, 61 vessels were recorded arriving in Fildes Bay, a third of the ships visiting the entire Antarctic continent^12^. Although ships were registered in 27 countries from all other continents (Supporting Information Table S3), 56% of these ships declared having sailed directly from ports in the Strait of Magellan and Beagle Channel sectors, respectively (Fig. 2). The subsequent destination of 84% of the ships was to points further south in Antarctica. Ship activity was limited to the October-March period and peaked around the Austral summer solstice. Over 50% of ship activity was associated with tourism, over a third with scientific research, and the remainder divided among cargo, military and fisheries operations (Fig. 2).

### Temperature in donor and recipient regions

The number of vessel visits is highest during peak summer water temperatures (up to 2.0 °C) in Fildes Bay (Fig. 2). In winter, temperature remained generally below −1 °C for over four months and occasionally was close to the freezing point (minimum: −1.9 °C). In comparison, temperatures from the Strait of Magellan varied from 5.9 to 9.9 °C in the subtidal (2017–2019 data) and from −1.4 and 21.7 °C in the intertidal (2017–2018, Fig. 2). In the intertidal, extreme values were recorded at low tide (i.e., emerged temperature) for a few hours a day.

## Discussion

The early detection of a species outside of its previous known distribution, including both native and invaded areas, triggers a series of cascading questions as to (1) the source of propagules (i.e., the donor region), (2) the mechanism by which they were transported and released into the recipient region, (3) the probability that the species will become established and finally (4) the possible impacts it will have. Here we have provided strong evidence for the first two questions and intriguing observations with regards to the latter two.

With regards to the source of the mussels that we discovered, we were able to assign all sequenced individuals to the southern hemisphere lineage of *Mytilus* sp., and more specifically to the S1 clade^20^. Although there is no consensus for the species name of this clade and it is not the scope of this study to deal with taxonomic questions, we suggest that this clade S1 could be referred to as *Mytilus* cf. *platensis* d’Orbigny, 1842^21–23^. Due to the relatively long planktotrophic larval period for mussels (up to 6 weeks^24^), and rafting ability^25^, mytilids would certainly have the potential to naturally disperse this distance. Indeed, in the late Tertiary era this clade was able to spread 3500 km to the Kerguelen Islands in the southern Indian Ocean, likely aided by strong eastward currents^23,26,27^. However, the position and strength of the Antarctic Polar Front was quite different at that time^20,23^, and it now represents a formidable oceanic barrier to poleward migration from South America and nearby islands^8,10^. For instance, passive dispersal from these locations towards islands of the Antarctic Peninsula likely lasts 1 to 3 years^27^, which may explain the absence or paucity of living non-indigenous biota observed upon biodegradable rafts (kelp) reaching Antarctic shores^13,27^.

Although a number of vectors can transport NIS propagules, the limited presence and activities of humans in the Antarctic make ship traffic the most likely vector^12^ for the mussels that we observed, especially given that most ship traffic either originates or passes through the most probable source population, that of southern Patagonia. However, the exact mechanism is not obvious as mussels can be transported either as adults attached to the hull, especially in the sea chest where fouling can be higher^14^, or as larvae in ballast water^28^. If ballast water was the mechanism, then larvae were certainly partially developed when released, but still would have had to complete development, settle and metamorphose. However, it is technically illegal to release ballast water in the nearshore environments of Antarctic (IMO Ballast Water Management Convention, Resolution MEPC.163(56)), which supports the idea that the juveniles were instead the result of adults spawning after the arrival of a fouled vessel^29^, the probability of which being exacerbated during the warmer period (our results, Fig. 2). If true, then it would mean that at least some of the resulting zygotes were able to complete the entire larval phase as well as settle and metamorphose. Given that the presence of adult *Mytilus* spp. on ships visiting Antarctica has already been repeatedly documented^14^ (specimens originating from South Africa were identified as *M. galloprovincialis* although the authors apparently did not conduct genetic analyses) in contrary to other vectors (e.g. plastic and kelp rafts)^13^, but see^25^, we suggest that this latter mechanism was the probable means by which the mussels we observed were released into Antarctic waters.

Regardless of the precise mechanism, the presence of juvenile mussels in Antarctica clearly demonstrates that the initial steps in the invasion process^17^, namely the transport and release of propagules of non-indigenous species, are occurring in this isolated ecosystem. Moreover, it shows that larvae are able to survive and recruit in this new system and demonstrates that environmental filters (e.g., mismatch between donor and recipient environments^17^) did not prevent key early phases of the life cycle (e.g., larval development, settlement and metamorphosis, and early juvenile growth) from occurring. This finding is remarkable as there is to the best of our knowledge no indication in the literature that mussel larval development can occur at the consistently low temperatures that characterize Antarctica. Following the mussel settlement herein reported, two key questions remain: will they stay, and if they do, what impact will they have?

“Staying” in this context means establishing a self-sustaining population, which would require both further individual growth and reproduction (gonad development and gametogenesis). Studies of Arctic populations show that gametogenesis and spawning can occur in populations of mytilid mussels at the low temperatures that we recorded in this study^30,31^, and, as argued above, the presence of juveniles in several locations far from ship activity is strong evidence for a successful passage from fertilization through larval development to settlement and metamorphosis in these very low temperatures. Finally, growth clearly has occurred as the observed shell length of 2 mm is far larger than the size at settlement for *M. platensis* recruits^32^. Information from this study demonstrates that the early parts of the mussel’s life cycle could be completed in Antarctic waters, which is consistent with observations of populations of *Mytilus* elsewhere where low temperatures are normal (e.g. as experienced by intertidal mussels in the Strait of Magellan, Fig. 2), albeit not as continuously low as recorded in Fildes Bay (i.e., <2 °C). However, at the time of this writing, we cannot ascertain the fate of the Fildes Bay mussel population, as the austral summer campaign of 2020 is still ongoing. Preliminary sampling and examination within the very same subtidal habitats suggest that this population has gone extinct locally, although further work in this area is, however, clearly needed to assess the risk of establishment in this environment.

Successful completion of the life cycle is a necessary but not sufficient condition for the establishment of a NIS as demographic limitations (e.g. Allee effect) are also a consideration. This concern is especially relevant to marine invertebrates like mussels that have separate sexes, external fertilization and long planktonic larval periods. This suite of characteristics leads to the dilution of gametes and larvae, which can limit rates of fertilization and recruitment, respectively, and together reduce the probability of establishing a self-sustaining population^33^. Indeed, several past cases of the appearance of mussel populations outside their known range illustrate the precarious nature of the invasion process. One example was the brief appearance of the temperate species *M. galloprovincialis* in a subtropical location (Hawaii, USA) where ship-fouling mussels spawned and recruited within a harbor but did not persist^29^. Another example was the appearance of *Mytilus* cf. *edulis*^34^ in Svalbard (Norway) due to an extreme alteration of oceanic circulation^35^. The first recruitment was estimated to have occurred in 2002, and subsequent sampling in 2014 confirmed the persistence of this population – likely aided by further inoculation events^34^. These contrasting examples demonstrate both the ephemeral nature of populations established outside their existing range as well as the need for both early detection efforts and frequent monitoring to ascertain the conditions under which such invasions succeed or fail.

Mussel invasions have been frequent worldwide. Impacts have been varied, but given the general importance of mussels in structuring benthic marine and freshwater communities^36^, the potential is enormous. One peculiarity of the present case is the occurrence of mussels associated with biogenic structures, such as sponges. Should the mussel remain a rare part of this cryptofauna, its visible impact will likely be negligible. In contrast, should this ecological niche provide a beachhead for spread into other habitats (e.g. notably in the intertidal, which have not presently been sampled but where densest mussel populations are commonly found elsewhere), then impacts could increase. Although the initial steps of colonization may be hampered by a loss of facilitating taxa (e.g. barnacles), *Mytilus* have the potential to spread in a broad arrays of environments and habitats, ranging from diversified submerged moving vectors^25,37^ to depauperate and ice-influenced intertidal shores^34^. At the extreme, the formation of extensive beds as observed in both indigenous and invaded regions^38^ could have major impacts on native biodiversity, food web dynamics and ecosystem processes (e.g., nutrient dynamics)^3,39^.

Lurking behind this discussion are two factors that will continue to redefine the probability of NIS establishment in Antarctica: global transportation and climate change^8,40,41^. For the former, both the volume and the pathways of ship traffic will increase over time as scientific research, commercial interests and tourism increase^12^. Certainly, measures can be put in place to reduce the probability of the transport of NIS propagules^15^. Whereas ballast water regulations have already been instituted under the Antarctic Treaty and International Maritime Organization, no international biosecurity measure for hull fouling (e.g. cf. Resolution MEPC.207(62)^42^) has entered into force yet in spite of strongly acknowledged risk for invasions in the Antarctic^19^. Nevertheless, the increased numbers of ships will inevitably lead to additional releases including in areas that are currently rarely visited. More worrisome, however, is climate change as the extreme conditions of Antarctic marine waters (water temperatures always <2 °C) represent a significant physiological barrier for species originating from more temperate regions. As temperatures in Southern Ocean increase^43^, more and more species will be able to establish and those that establish will have greater and greater impacts. Indeed, even small changes in water temperatures can have large effects^44^, and the most worrisome element of the information presented in this study is not that this particular population of mussels is poised to impact the benthic communities of Antarctica, but rather it provides a glimpse of “invasions future”: the vector has been identified, the pathways have been described and at least one of the environmental filters has failed. The ship traffic data have shed light on putative vectors and pathways of introduction and shown a strong potential of propagule and colonization pressures^45^ of Patagonian taxa. Moreover, Fildes Bay and other locations in the South Shetland Islands may become important stepping stones for further spread in Antarctica. Finally, completion of the larval period in such low temperatures represent a major step in the invasion process. Whether spawning occurred *in situ* from transported adults (e.g. on vessel hulls or in sea chests) or within their native range, followed by larval transport (e.g. within ballast tanks) cannot be ascertained^12,14^, but in any case, barriers may be weakening and the bar for invasion may be much lower than believed. The Antarctic constitutes a distinct biogeographic realm, and global warming will not only put charismatic native species at risk, but also lower dispersal and physiological barriers to NIS in intertidal and shallow waters^18,27^. Whether a result of direct anthropogenic causes or indirect biotic interactions, the resulting changes in the unique biotic assemblages of Antarctica will represent an irreversible change to one of the most unique marine biotas on the planet.

## Methods

### Biotic sampling

Samples were collected in the austral summer of 2019 from the surface of subtidal vertical walls at 2 sites separated by 3 km in Fildes Bay (King George Island, Antarctica; Supporting Information, Fig. S2) as part of a biodiversity survey. Here, the subtidal seascape is characterized by emergent rocks and boulders colonized by diverse communities of invertebrates and macroalgae. Benthic assemblages between 5 and 15 m depth were sampled by SCUBA divers from a series of vertical walls (six at Site 1 and two at Site 2) each 10–15 m long and separated by >30 m. On each wall, a total of 10 photo-quadrats (separated by >1 m) were taken to determine the percent cover of the larger taxa (e.g. sponges, cnidarians, seaweeds). All sessile organisms were scraped from a series of circular cores (10 cm in diameter and often up to 14 cm thick, n = 5–10 per seawall) and collected into a 1-mm-mesh bag). Samples were transported directly to the laboratory in the nearby Chilean Antarctic station “Prof. Julio Escudero”. There they were sorted and identified to the lowest possible taxonomic level within 24 hours. All mussels found were immediately preserved in 95% EthOH, and shell lengths measured later using scanning electronic microscopy at the Universidad Austral de Chile (Valdivia, Chile).

### Mussel identification

Because the mussels could only be morphologically identified to the genus *Mytilus*, we used molecular barcoding for a more precise identification. Genomic DNA was extracted from the soft tissue of 15 mussels using the Zymo Quick-DNA Universal Kit (Zymo Research) following the manufacturer’s protocol. For each sample, DNA concentration and purity were analyzed in a nanodrop 2000 (ND2000 Thermo Scientific) and its integrity was verified on a 1.2% denaturated agarose gel. The molecular markers were the mitochondrial 16 S ribosomal DNA (16 S rDNA) using the primers 16S-ar and 16S-br^46^ and the mitochondrial cytochrome c oxidase subunit I (COI) using the primers LCO1490 and HCO2198^47^. PCRs were performed using a thermocycler (Multigene Optimal, Labnet) following Gérard *et al*.^20^. Amplicons were sequenced by the Sanger method using an ABI PRISM 3100 Genetic Analyser at the core facility of Universidad Austral de Chile (www.australomics.cl).

To evaluate the genetic relationship of the mussels discovered in Antarctica to possible source populations, our sequences were compared to 16S rDNA and COI sequences obtained from the National Center for Biotechnology Information database (https://www.ncbi.nlm.nih.gov) for *Mytilus* specimens from other regions of the world (Details in Supporting Information Tables S1 and S2; see also^20^. To complement this dataset, mussels were collected from intertidal riprap near Punta Arenas (53.096776°S, 70.872978°E), a likely source population of these mussels (see results), and sequenced as described above (the genetic data for the Antarctic and Punta Arenas specimens were deposited in the Genbank database with the accession numbers MN696175-201). Sequences alignments were carried out using Geneious Pro^48^. Since the sequences available in the NCBI database were shorter than ours, we used a database of 105 sequences of a total length of 404 bp of the 16S rDNA gene and 179 sequences of a total length of 400 bp of the COI gene (details in Supporting Information Tables S1 and S2). First, phylogenetic reconstructions for both genes were performed using neighbor-joining distance (NJ) and maximum-likelihood (ML) approaches using the software Mega^49^. Levels of significance of tree nodes were determined using 1000 bootstrap replications. To determine the spatial genealogical relationship among populations, a haplotype network was constructed for the southern hemisphere lineage of *Mytilus* sp. using 103 COI sequences with a total length of 632 bp following the procedure described by Salzburger, *et al*.^50^.

### Vectors and pathways

Since ship traffic is by far the most likely vector for transporting mussels to Antarctica^12,40^, we assessed the shipping activity in Fildes Bay to determine possible pathways of introduction. We used data gathered by the Chilean navy (based within Fildes Bay yearlong) over the most recent two-year period (2017–19) for each stopover in the bay (n = 112–152 per yr^−1^). The information obtained consisted of (1) arrival date, (2) ship name, (3) type of activity, (4) administrative base, (5) last port of call, and (6) intended destination. Geographic data for origins and destinations were then grouped into ten sectors: temperate South America, tropical South America, South Africa, eastern Patagonia, Strait of Magellan, Beagle Channel, Falkland Islands, South Georgia and the South Sandwich Islands, other South Shetland Islands, and other Antarctica locations (unfortunately, precise geographic information was not available for other Antarctic origins and destinations, which were typically (>95% of reports) referred to simply as “Antarctica” by the controllers). Over the full two-year period, temporal trends of shipping activity were examined as the number of ships arriving in Fildes Bay each month.

### Temperature in donor and recipient regions

We compared seawater temperatures from Fildes Bay with southern Patagonia, the closest region where indigenous populations of *Mytilus* sp. occur (Fig. 1). Southern Patagonian sites were located in the Strait of Magellan (Faro San Isidro, 53,68444°S, 70,99166°W) and in the Beagle Channel (54,9175°S, 64,64673°W). At Faro San Isidro, temperature was measured at 15 minutes intervals between February 2017 and December 2018 using data loggers (DST CT, Star Oddi ®) deployed in the subtidal (at 10-m depth) and the mid-intertidal zones. Loggers were recovered and replaced in February 2018, but after the second sampling period (2018–2019), the intertidal logger could not be found. In the Beagle Channel, temperature data were obtained from hourly measurements at a 50-m depth using a DST CT logger from July 2017 to July 2018. Finally, temperature data from Fildes Bay (62,19439°S, 58,9174°W) were also obtained from *in situ* measurements at 30 minutes intervals at 10-m depth using a DST CT logger between February 2017 and January 2019.

## Supplementary Information

Supplementary Information.
