# Supplementary material for: First mussel settlement observed in Antarctica reveals the potential for future invasions
Source: Sci Rep. 2020 Mar 26;10:5552. doi: 10.1038/s41598-020-62340-0 (PMC7099062; doi:10.1038/s41598-020-62340-0)
Supplement: Supplementary file 1 — Supplementary Information. [file 41598_2020_62340_MOESM1_ESM.docx]

**Supporting Information**

Ghosts of invasion future and first mussel settlement observed in Antarctica

Leyla Cárdenas^1*, 2†^, Jean-Charles Leclerc^3†^, Paulina Bruning^1,6^, Ignacio Garrido^1, 6^, Camille Détrée^1^, Alvaro Figueroa^1,2^ , Marcela Astorga^4^ , Jorge M. Navarro^1,5^ , Ladd E. Johnson^6^ , James T. Carlton^7^ & Luis Pardo^1,5^

**Institutional Affiliation**

^1^Centro FONDAP de Investigación en Dinámica de Ecosistemas Marinos de Altas Latitudes (IDEAL), Chile.

^2^ Instituto de Ciencias Ambientales y Evolutivas, Facultad de Ciencias, Universidad Austral de Chile, Valdivia, Chile.

^3^Universidad Católica de la Santísima Concepción, Centro de Investigación en Biodiversidad y Ambientes Sustentables (CIBAS), Concepción, Chile.

^4^Instituto de Acuicultura, Universidad Austral de Chile, Puerto Montt, Chile

^5^Instituto Ciencias Marinas y Limnológicas, Facultad de Ciencias, Universidad Austral de Chile, Valdivia, Chile

^6^Department of Biology and Quebec-Ocean Institute, Laval University, Québec, QC, Canada

^7^Maritime Studies Program, Williams College, Mystic, CT, USA

*Contact Information: Leyla Cárdenas

Phone number: +56 63 221673, Email: leylacardenas@uach.cl

^†^These authors contributed equally to this work.

**This file includes:**

**Figure S1.** ML Phylogenetic relationships based on 16S rDNA and COI haplotypes in *Mytilus* spp*.* mussels. Bootstrap scores larger than 50% are indicated on branches for NJ and ML, respectively. Genbank accession numbers are given in the tips of the branch. See Tables S1 and S2 for details. Trees were rooted with *Mytilus trossulus.* Antarctic samples are marked in red.

**Figure S2**. Location of the sampling area in Fildes Bay, King George Island, Antarctica. Map was depicted using the Ocean Data View Version: ODV 5.2.1 (Jan 30 2020). <https://odv.awi.de/>

**Tables S1 to S3**

**Table S1.** Details of 16S rDNA sequences obtained from the National Center for Biotechnology Information database (<https://www.ncbi.nlm.nih.gov>

**Table S2**. Details of COI sequences obtained from the National Center for Biotechnology Information database (<https://www.ncbi.nlm.nih.gov> )

**Table S3**. Compilated data of ships that arrived in Fildes Bay in a two-year period (2017-19)

**Table S1.** Details of 16S rDNA Sequences obtained from the National Center for Biotechnology Information database (<https://www.ncbi.nlm.nih.gov>)

| **Species** | **Locations** | **Code** | **N** | **GenBank Accession** | **Reference** |
| --- | --- | --- | --- | --- | --- |
| *Mytilus* sp. | Kerguelen islands | Ker | 2 | AM904589-AM904590 | Gérard et al (2008) |
| *M edulis* | no data |  | 1 | AF317054 | Barna & Showman, Unpublished |
| *M edulis* | no data |  | 1 | AJ293738 | Quesada et al. Unpublished |
| *M edulis* | DE, USA | USA | 1 | U22866 | Rawson & Hilbish (1995) |
| *M edulis* | no data |  | 1 | AF317055 | Barna & Showman, Unpublished |
| *M edulis* | DE, USA | USA | 2 | AJ293730-AJ293738 | Quesada et al. Unpublished |
| *M edulis* | DE, USA | USA | 4 | AF023546-AF023547-AF023549-AF023551 | Rawson & Hilbish (1995) |
| *M edulis* | DE, USA | USA | 1 | KC429249 | Sharma et al. (2013) |
| *M edulis* | Bergen, Norway | Nor | 3 | U22866-U22867-U22868 | Rawson & Hilbish (1995) |
| *M edulis* | Bergen, Norway | Nor | 1 | GQ455405 | Westfall et al. (2010) |
| *M. galloprovincialis* | No data |  | 1 | AF317056 | Barna & Showman, Unpublished |
| *M. galloprovincialis* | Morocco, Africa | AFR | 2 | KT021638–39 | Astorga et al. (2015) |
| *M. galloprovincialis* | Australia | Aus | 8 | GQ472141-GQ472142-GQ472143-GQ472144-GQ472145-GQ472151-GQ472152-GQ472153 | Liu et al. (2011) |
| *M. galloprovincialis* | Japan | JP | 20 | KC835224-KC835225-KC835226-KC835227-KC835228-KC835229-KC835230-KC835231-KC835232-KC835233-KC835242-KC835243-KC835244-KC835245-KC835246-KC835247-KC835248-KC835249-KC835250-KC835251 | Brannock et al. (2013) |
| *M. galloprovincialis* | South Hemisphere |  | 1 | GQ455398 | Westfall et al. (2010) |
|  | China | Cha | 1 | GQ472141 | Lui & Li 2009, Unpublished |
|  | China | Cha | 1 | GQ472153 | Lui & Li 2009, Unpublished |
| *M. galloprovincialis* | Chile | Ch | 1 | KP052902 | Astorga et al (2015) |
| *Mytilus sp.* | New Zealand | NZ | 2 | AM904568-AM904569 | Gérard et al (2008) |
| *M. galloprovincialis* | Tasmania | TAS | 2 | AM904579-AM904580 | Gérard et al (2008) |
| *Mytilus sp.* | Wellington Harbour | NZ | 2 | AM904570-AM904572 | Gérard et al (2008) |
|  | Kerguelen islands | Ker | 2 | AM904589-AM904590 | Gérard et al (2008) |
| *M. galloprovincialis* | Australia | AUS | 1 | AF179448 | Hilbish et al. (2000) |
| *M. galloprovincialis* | Kerguelen islands | Ker | 1 | AF179449 | Hilbish et al. (2000) |
| *M. galloprovincialis* | New Zealand | NZ | 2 | AF179452-AF179453 | Hilbish et al. (2000) |
| *M. galloprovincialis* | Australia | Aus | 3 | AM904592-AM904593-AM904594 | Gérard et al (2008) |
| *M. trossulus* | Japan | JP | 2 | KC835211-KC835213 | Brannock et al. (2013) |
| *Mytilus chilensis* | Punta Arenas | Ch | 25 | KP052860-KP052885 | Astorga et al (2015) |
| *Mytilus chilensis* | Uruguay | Uru | 7 | KR153827–33 | Astorga et al (2015) |

**Table S2.** Details of COI Sequences obtained from the National Center for Biotechnology Information database (<https://www.ncbi.nlm.nih.gov>)

a) For Phylogenetic Analysis

| **Species** | **Locations** | **code** | **N** | **Genebank Accession** | **Reference** |  |
| --- | --- | --- | --- | --- | --- | --- |
| *M planatus/platensis* | Chile | CHI | 19 | KR066743-52,68, KR6668, KR066717, KR66723 | Astorga et al. 2015 | |
| *M planatus/platensis* | Argentina | ARG | 6 | KR066758-63 | Astorga et al. 2015 | |
| *M planatus/platensis* | Uruguay | URU | 4 | KR066764-67 | Astorga et al. 2015 | |
| *M chilensis* | Chile | CHI | 2 | AM905195-5186 | Gerard et al. 2008 | |
| *M galloprovinciallis* | Kerguelen; fr | KER | 5 | AM905211 | Gerard et al. 2008 | |
| *M galloprovinciallis* | Canada | CAN | 1 | DQ864416 | Lewis & Jarman 2006 | |
| *M galloprovinciallis* | New Zealand | NZ | 10 | DQ864378-84 | Lewis & Jarman 2006 | |
| *M galloprovinciallis* | Australia | AUS | 4 | AM905161;62;65,68 | Gerard et al. 2008 | |
| *M galloprovinciallis* | Australia | AUS | 19 | DQ864378–96 | Lewis & Jarman 2006 | |
| *M galloprovinciallis* | Australia | AUS | 15 | DQ86411–25 | Lewis & Jarman 2006 | |
| *M galloprovinciallis* | New Zealand | NZ | 3 | AM905146;49, 55 | Gerard et al. 2008 | |
| *M galloprovinciallis* | South Africa | AFR | 14 | DQ864397–410 | Lewis & Jarman 2006 | |
| *M edulis* | North Atlantic | NORATLA | 31 | AF241969–71 | Wares & Cummingham 2001 | |
| *M edulis* | Germany | GER | 23 | JF825556–689 | Steinert et al. 2012 | |
| *M trossulus* | North Atlantic | outgroup | 8 | AF242027-2035 | Wares & Cummingham 2001 | |

B) For Phylogeographic analysis. Only samples from South America, Kerguelen, Australia and New Zealand.

| **Locations** | **N** | **Code** | **GenBank Accession** | **References** |
| --- | --- | --- | --- | --- |
| Cloudy Bay lagoon, Tasmania | 4 | Aus | AM905161-AM905162-AM905163-AM905164 | Gérard et al (2008) |
| Simpson’s Bay, Tasmania | 3 | Aus | AM905165-AM905166-AM905167 | Gérard et al (2008) |
| Hobart, Tasmania | 6 | Aus | AM905168-AM905169-AM905170-AM905171-AM905172-AM905173 | Gérard et al (2008) |
| Maullin, Chile | 7 | Ch | AM905180-AM905181-AM905182-AM905183-AM905184-AM905185-AM905186 | Gérard et al (2008) |
| Patagonia, Chile | 9 | Ch | AM905187-AM905188-AM905189-AM905190-AM905191-AM905192-AM905193-AM905194-AM905195 | Gérard et al (2008) |
| Kerguelen islands | 16 | Ker | AM905196-AM905197-AM905198-AM905199-AM905200-AM905201-AM905202-AM905203-AM905204-AM905205-AM905206-AM905207-AM905208-AM905209-AM905210-AM905211 | Gérard et al (2008) |
| Punta Arenas, Chile | 21 | Ch | KR066728-KR066729-KR066730-KR066731-KR066732-KR066734-KR066735-KR066736-KR066737-KR066738-KR066739-KR066740-KR066741-KR066745-KR066746-KR066747-KR066748-KR066749-KR066750-KR066751-KR066752 | Astorga et al. 2015 |
| Wellington Harbour, New Zealand | 6 | NZ | AM905149-AM905150-AM905151-AM905152-AM905153-AM905154 | Gérard et al (2008) |
| Dunedin, New Zealand | 3 |  | AM905146-AM905147-AM905148 | Gérard et al (2008) |
| George Sound, New Zealand | 6 | NZ | AM905155-AM905156-AM905157-AM905158-AM905159-AM905160 | Gérard et al (2008) |

**Table S3**. Compilated data of ships that arrived in Fildes Bay in a two-year period (2017-19).

| Main activity | Ship code | Region | Country | 2017-2018 | 2018-2019 |
| --- | --- | --- | --- | --- | --- |
| Tourism | Ship#29 | South America | UK | 5 | 1 |
| Tourism | Ship#38 | South America | Chile | 5 | 9 |
| Tourism | Ship#14 | Oceania | New Zealand | 1 | 1 |
| Tourism | Ship#22 | Oceania | Marshall Islands |  | 5 |
| Tourism | Ship#26 | Oceania | Marshall Islands | 2 |  |
| Tourism | Ship#37 | North America | USA |  | 1 |
| Tourism | Ship#54 | North America | USA | 2 |  |
| Tourism | Ship#13 | Europe | Netherlands |  | 1 |
| Tourism | Ship#23 | Europe | Norway |  | 1 |
| Tourism | Ship#27 | Europe | UK | 1 |  |
| Tourism | Ship#34 | Europe | Netherlands |  | 1 |
| Tourism | Ship#42 | Europe | Norway |  |  |
| Tourism | Ship#43 | Europe | France | 1 |  |
| Tourism | Ship#49 | Europe | Chypre |  | 1 |
| Tourism | Ship#50 | Europe | UK |  | 1 |
| Tourism | Ship#51 | Europe | Netherlands | 1 |  |
| Tourism | Ship#52 | Europe | Malta | 2 |  |
| Tourism | Ship#57 | Europe | Malta |  | 3 |
| Tourism | Ship#20 | Caribbean | UK |  | 6 |
| Tourism | Ship#21 | Caribbean | UK | 1 |  |
| Tourism | Ship#28 | Caribbean | Bahamas | 1 |  |
| Tourism | Ship#30 | Caribbean | Antigua and Barbuda | 3 | 4 |
| Tourism | Ship#31 | Caribbean | Bahamas | 2 | 2 |
| Tourism | Ship#32 | Caribbean | Caiman Islands |  | 1 |
| Tourism | Ship#40 | Caribbean | Panama | 4 | 3 |
| Tourism | Ship#45 | Caribbean | Bahamas | 3 | 4 |
| Tourism | Ship#46 | Caribbean | Bahamas |  | 1 |
| Tourism | Ship#47 | Caribbean | Bahamas | 12 | 15 |
| Tourism | Ship#48 | Caribbean | St Vincent and the Grenadines | 3 | 4 |
| Tourism | Ship#59 | Caribbean | Bahamas | 1 | 1 |
| Tourism | Ship#53 | Asia | Russia | 1 | 4 |
| Tourism | Ship#60 | Africa | Comoros |  | 3 |
| Scientific | Ship#4 | South America | Argentina |  | 1 |
| Scientific | Ship#5 | South America | Brazil | 2 | 1 |
| Scientific | Ship#9 | South America | Argentina | 1 | 1 |
| Scientific | Ship#10 | South America | Argentina |  | 1 |
| Scientific | Ship#11 | South America | Argentina |  | 3 |
| Scientific | Ship#12 | South America | Columbia | 3 | 2 |
| Scientific | Ship#15 | South America | Peru |  | 4 |
| Scientific | Ship#18 | South America | Argentina |  | 2 |
| Scientific | Ship#19 | South America | Peru | 1 |  |
| Scientific | Ship#35 | South America | Chile | 1 | 10 |
| Scientific | Ship#44 | South America | Brazil | 3 | 7 |
| Scientific | Ship#56 | South America | Uruguay |  |  |
| Scientific | Ship#17 | Europe | Spain | 1 | 4 |
| Scientific | Ship#33 | Europe | UK |  | 1 |
| Scientific | Ship#36 | Europe | Poland |  | 1 |
| Scientific | Ship#58 | Europe | Spain | 5 |  |
| Scientific | Ship#2 | Asia | Russia |  | 3 |
| Scientific | Ship#3 | Asia | Russia | 2 | 2 |
| Scientific | Ship#1 | Africa | South Africa | 1 |  |
| Military | Ship#8 | South America | Chile | 2 | 3 |
| Military | Ship#24 | South America | Chile | 5 | 8 |
| Military | Ship#25 | South America | Chile |  | 1 |
| Military | Ship#61 | South America | Chile |  | 5 |
| Military | Ship#39 | Europe | France | 1 |  |
| Fishery | Ship#6 | South America | Chile |  | 3 |
| Fishery | Ship#7 | South America | Chile | 2 | 4 |
| Cargo | Ship#16 | South America | Chile | 3 | 4 |
| Cargo | Ship#55 | South America | Uruguay | 1 |  |
| Cargo | Ship#41 | Asia | Kuwait | 2 | 1 |

Figure S1

Figure S2
